# A Paternally Inherited *BRCA1* Mutation Associated with an Unusual Aggressive Clinical Phenotype

**DOI:** 10.1155/2014/875029

**Published:** 2014-02-10

**Authors:** Florentia Fostira, Nikolaos Tsoukalas, Irene Konstantopoulou, Vassilios Georgoulias, Charalambos Christophyllakis, Drakoulis Yannoukakos

**Affiliations:** ^1^Molecular Diagnostics Laboratory, INRASTES, NCSR Demokritos, 15310 Athens, Greece; ^2^Medical Oncology Unit, “401” General Military Hospital, 11525 Athens, Greece; ^3^Department of Medical Oncology, University General Hospital of Heraklion, 71110 Crete, Greece

## Abstract

This report highlights the necessity of genetic testing, at least for *BRCA1* mutations, of young females diagnosed with triple negative breast cancer, even in the absence of or limited family history. A 34-year-old female with a locally advanced, triple negative tumour, which perforated the skin, is described. At the time of diagnosis, the patient had already multiple lung metastases and although chemotherapy was started immediately, she died with rapid systemic disease progression. The patient was found to carry the *BRCA1* p.E1060X mutation, which is located on exon 11 of the gene. The high penetrance of *BRCA1* gene is not represented in the patient's family, since the mutation was paternally inherited. It is evident that females belonging to small families, along with paternal inheritance of pathogenic *BRCA* mutations that predispose for breast cancer, in most cases will probably be genetically tested only after being diagnosed with cancer.

## 1. Introduction

*BRCA1* and *BRCA2* genetic testing has been available in the clinic at least for the past decade, allowing the characterization of people that face an increased breast and ovarian cancer risk. Although the selection criteria for genetic testing are quite established, many times the small size of the family or the paternal inheritance of the pathogenic *BRCA *mutation can be misleading when referring patients. Therefore, loss-of-function mutations can be genetically transmitted from male *BRCA* mutation carriers, who can be in many cases cancer-free, to their daughters, who will have a lifetime breast cancer risk that can be as high as 84% [1–3]. Estimated lifetime breast cancer risk for male *BRCA2* carriers is approximately 8% [4], while there is high relative risk for pancreatic and prostate cancer, when compared to the general population [5]. On the contrary, relative and cumulative cancer risks are much lower in male *BRCA1* carriers [6]. Since our current knowledge is advanced on understanding the cancer predisposition of mutation carriers, it seems rather important to successfully identify these individuals in order to offer appropriate clinical management.

## 2. Case Report

A 34-year-old premenopausal female with a locally advanced tumour presented as a dirty ulcer, perforating the skin, in her right breast is described. Biopsies of the damaged tissue showed an invasive, grade III ductal carcinoma, with areas of papillary shaping, areas of necrosis, and invasion of the skin. The breast tumour was classified as triple negative, since there was a lack of estrogen and progesterone receptor expression and absence of HER2 oncoprotein after immunohistochemical staining. Staging revealed multiple lung metastases and enlargement of right axillary lymph nodes. Consequently, she was diagnosed with a stage IV infiltrating ductal cancer in her right breast which was triple negative and grade III.

The patient was started on first line chemotherapy with the regimen carboplatin 2AUC and paclitaxel 80 mg/m^2^ weekly plus bevacizumab 10 mg/Kgr on days 1 and 15. Initially she had a partial response to this treatment. However, her disease relapsed and she started on second line chemotherapy with the regimen docetaxel 75 mg/m^2^, adriamycin 60 mg/m^2^, and cytoxan 600 mg/m^2^ every 21 days. She received only two cycles of this chemotherapy because she was admitted to hospital due to multiple brain metastases. She was administered whole brain radiotherapy with minimal improvement of her clinical symptoms. Taking into account the triple negative, along with the *BRCA1* mutation status, the patient was considered as a candidate for entering a clinical trial with PARP inhibitors. Unfortunately, due to a rapid systemic disease progression the patient finally passed away a month later and therefore did not receive any further treatment.

**Figure 1:**
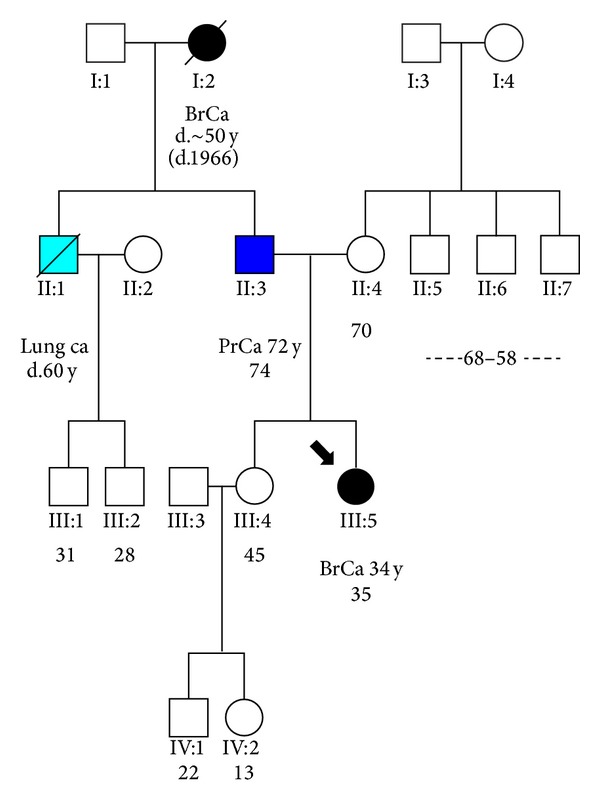
Pedigree of the family where the *BRCA1* mutation p.E1060X (c.3178G>T) was identified. The proband is highlighted by the arrow.

The proband had limited family history of breast or ovarian cancer. It is noteworthy that the family was rather small (Figure 1). Her grandmother from her father's side was diagnosed with breast cancer and died at her early fifties. The other reported cancer in her first degree relatives is the prostate cancer diagnosed in her father when he was 72. He underwent prostatectomy for prostate cancer, due to his elevated PSA value (7 ng/mL). The histology report revealed the existence of a low differentiated adenocarcinoma of the prostate gland with Gleason's pattern 7 (4+3). Also there were some areas with high grade prostatic intraepithelial neoplasia (PIN). The stage of the disease was T3bN0M0. Consequently, he was diagnosed with a high risk prostate cancer based on T3b. Initially, he received radiotherapy and hormonal therapy with LHRH analogue and specifically triptoreline and antiandrogen (bicalutamide).

Many research groups have reported that *BRCA1* and *BRCA2* mutation carriers are more likely to develop a more aggressive prostate cancer phenotype, generally associated with a higher probability of nodal involvement, distant metastasis, and low grade tumours [7, 8]. This observation is consistent with this case.

Due to the early age of breast cancer diagnosis, along with the distinct immunophenotype, the patient fulfilled the updated NCCN guidelines (http://www.nccn.org/) and was therefore tested for deleterious mutations in *BRCA1* and *BRCA2* genes. The direct relationship between *BRCA1* mutations and triple negative breast cancer has been widely assessed in Greek patients [9]. Direct Sanger sequencing revealed the nonsense mutation p.E1060X (c.3178G>T), which is located on exon 11 of the *BRCA1* gene. This is a rare, to the Greek population, mutation. The proband's father was tested to be the mutation carrier, while the proband's sister (cancer free at the age of 45) did not carry the damaging allele.

## 3. Discussion

This case report highlights the necessity of genetic testing even when family history is not prominent, at least for *BRCA1* mutations, of young females diagnosed with triple negative breast cancer. Individuals belonging in small families in combination with paternal inheritance of pathogenic mutations can remain undiagnosed. Some recent research studies indicate that females with paternally inherited mutations in *BRCA* gene mutations develop breast cancer at younger age when compared to women who inherit the gene mutations from their mothers. This can be partially explained by different imprinting patterns in maternal and paternal chromosomes and more specifically in the *BRCA1* or *BRCA2* locus [10].

We report this case as it highlights the importance of careful selection of individuals for genetic testing. The necessity for identification of mutation carriers is enlarged since there are promising, targeted therapies for *BRCA1* carriers, such as PARP inhibitors.
